# Biological sex influences severity and outcomes in *Acinetobacter baumannii* pneumonia

**DOI:** 10.1128/spectrum.03199-24

**Published:** 2025-04-16

**Authors:** Matthew S. Linz, Louis Collins, Emily Keenan, Ruchi Biswas, Dov Levine, Arun Mattappallil, Diana Finkel, Dane Parker

**Affiliations:** 1 Department of Pathology, Immunology and Laboratory Medicine, Center for Immunity and Inflammation, Rutgers New Jersey Medical School 12286, Newark, New Jersey, USA; 2 Division of Infectious Diseases, Department of Medicine, Rutgers New Jersey Medical School, Newark, New Jersey, USA; 3 Department of Surgery, Rutgers Robert Wood Johnson Medical School 12287 , New Brunswick, New Jersey, USA; 4 Department of Pharmaceutical Services, University Hospital 21655, Newark, New Jersey, USA; University of Melbourne, Melbourne, Australia

**Keywords:** *Acinetobacter baumannii*, sex bias, critical care medicine, bacterial pneumonia

## Abstract

Biological sex is known to affect the incidence and outcomes of infection, varying significantly by pathogen. The bacterial pathogen *Acinetobacter baumannii* is recognized as a serious global healthcare threat worldwide, yet understanding of how biological sex impacts its infection course is limited. We previously documented in a murine model of acute pneumonia that female mice were more susceptible to infection, but there are no published studies looking at outcomes in humans according to biological sex. We conducted a retrospective cohort study of 220 adult patients with a positive *A. baumannii* culture and clinical evidence of pneumonia. Contrary to the animal data, we observed male patients to have a longer overall (32 vs 24 days, *P* < 0.05) and intensive care unit (ICU) length of stay (LOS) (23 vs 17 days, *P* < 0.001) compared to females. The primary diagnosis at admission was only a factor when it was respiratory in nature. Younger male patients had higher Pneumonia Severity Index score on admission compared to younger females. Older males required more interventions than younger males. We also observed that mortality rates were increased for patients with chronic obstructive pulmonary disease (*P* = 0.024) and renal disease (*P* < 0.001), while alcohol use or smoking within 30 days prior to admission or recent surgery all negatively impacted LOS. This study highlights the importance of sex-and-gender-based studies, identifying worse outcomes in men, the elderly, and patients with certain underlying conditions and guiding efforts to improve management of patients with *A. baumannii* pneumonia.

**IMPORTANCE:**

Biological sex has been shown to influence the incidence and outcomes of infection. We had previously documented that in a mouse model of infection, the pathogen *Acinetobacter baumannii* caused more serious pulmonary disease in female animals. In this study, we aimed to determine if this was evident in human pneumonia data. We found that, opposite to the mice data, human males had extended hospital stays due to *A. baumannii* pneumonia. We also identified a number of risk factors that can impact mortality and duration of hospital stay. This information could be used to guide efforts to improve management of patients with *A. baumannii* pneumonia.

## INTRODUCTION

Biological sex refers to variations between males and females because of distinct sex chromosomes (1). Males and females mount different immune responses to infection and vaccines, while exhibiting different incidence rates to various autoimmune diseases and cancer. These responses are still a topic of active investigation, but are known to be mediated by sex chromosomes, hormones, nutrition, and the microbiome (2). Women have historically been underrepresented in clinical trials, creating a knowledge gap of how biological sex affects the incidence, prevalence, and severity of various infectious diseases (3–5). The relationship between sex and patient factors varies by pathogen; for instance, males have a higher prevalence of hantavirus infection, but females have increased disease severity (6–12). Sex bias also exists within infection types; for sexually transmitted bacterial infections, males have a higher prevalence and disease severity for gonorrhea (13–16), while females have higher prevalence and recurrence rates for chlamydia (13, 16, 17). These differences may have multifactorial causes, as some of these factors may be driven by gender-based behaviors rather than factors influenced solely by biological sex (18).

While sex differences in infectious disease are well recognized, understanding of the role of sex in infection is incomplete for many pathogens, including *Acinetobacter baumannii. A. baumannii* is a Gram-negative bacterium strongly associated with multidrug-resistant invasive infections, admission to the intensive care unit (ICU), with mortality rates as high as 40% to 60% (19–22). In 2018, *A. baumannii* was recognized as a critical priority pathogen by the World Health Organization (23). In 2019, *A. baumannii* was the leading bacterial cause of death in four countries, accounting for more than 452,000 deaths (24), and resistant *A. baumannii* alone was associated with more than 300,000 deaths (25). Despite the recognition of *A. baumannii* infections as a serious global healthcare threat, understanding how biological sex impacts their course in humans is limited.

A recent study from our group determined that female C57BL/6J mice had increased susceptibility to *A. baumannii* pneumonia (26). Infected female mice had a decreased survival rate and bacterial counts that were significantly higher in bronchoalveolar lavage fluid (BALF), lung tissue, and the spleen 24 h after infection compared to male mice. There are a few studies in humans showing that males are more likely to develop infection with *A. baumannii* than females (27–29). However, to our knowledge, there are no published studies evaluating the severity of patient symptoms or outcomes after infection. Given the limited human data, we sought to expand on our animal studies and determine if there was a sex bias in *A. baumannii* pneumonia in human patients by retrospectively reviewing demographic data and clinical outcomes from the electronic medical record (EMR) at our affiliated teaching hospital, University Hospital in Newark, New Jersey, USA.

## MATERIALS AND METHODS

### Setting and participants

**Fig 1:**
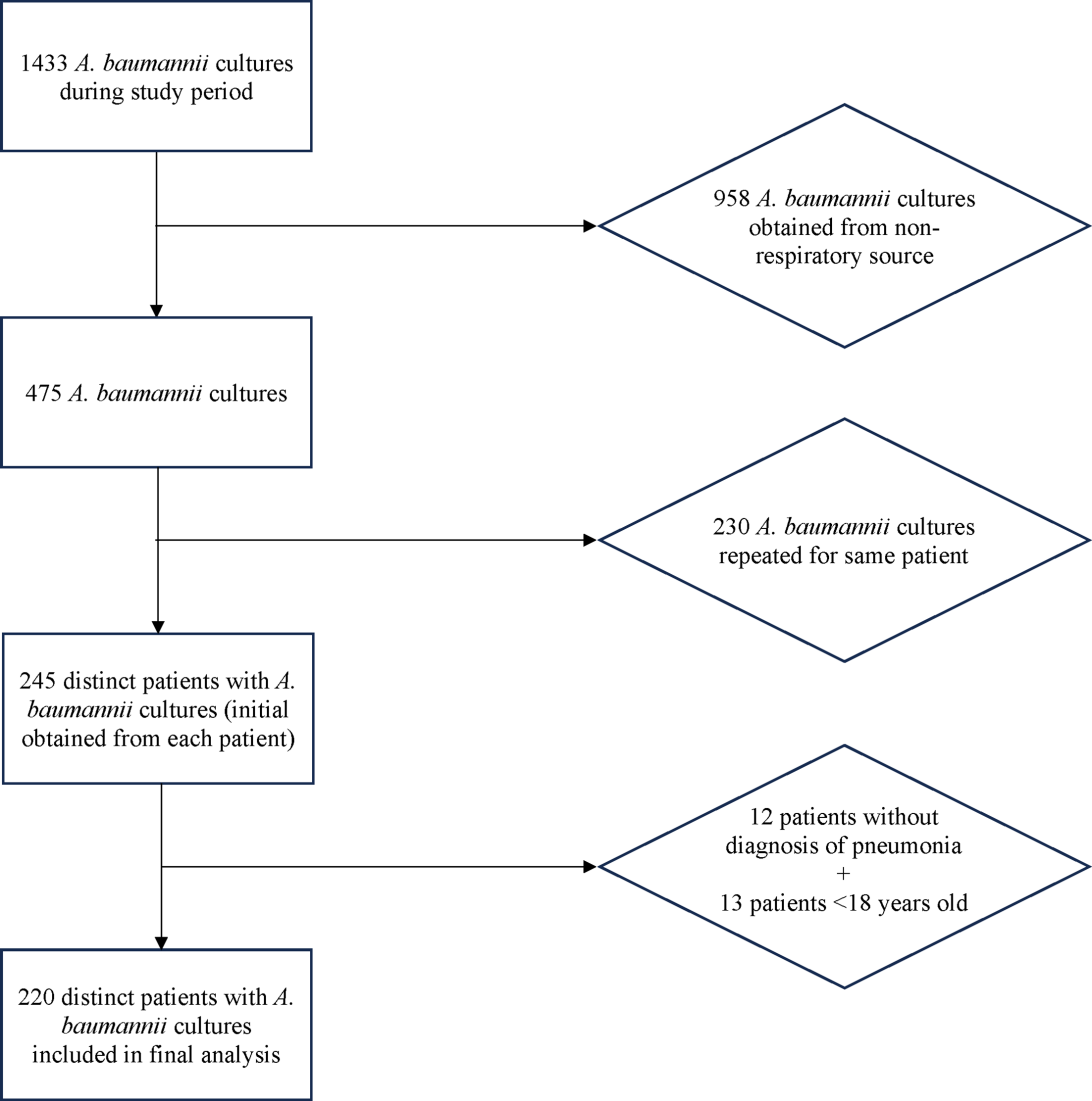
Inclusion and exclusion criteria.

This study was conducted for patients with a positive *A. baumannii* culture at the University Hospital in Newark, New Jersey, USA, a tertiary-care academic center. Patients were separated by biological sex as reported in the electronic medical record. We retrospectively reviewed all positive respiratory cultures obtained from patients, between 1 November 2016 and 1 November 2022, who had clinical evidence of pneumonia as coded or as per physician documentation. Only the first positive culture was included for each individual patient; we excluded any patients younger than 18 or without documented clinical evidence of pneumonia (Fig. 1).

### Definitions, measures, and outcomes

We classified each patient into a primary diagnosis admission group (respiratory, neurologic, gastrointestinal, etc.). Demographic measures included age, race, ethnicity, intubation, and ventilation status during admission. We also looked at co-infection status and antimicrobial resistance patterns, using the international definition of multi-drug resistance (MDR) as non-susceptibility to at least one agent in three or more antimicrobial categories (30). We recorded patient comorbidities and risk factors including a history of MDR organisms, recent surgery, smoking, alcohol use, and intravenous drug use within 30 days of admission as well as prior antimicrobial use, prior hospitalization, and admission to a nursing home or other long-term acute care facility within 90 days of admission. Outcomes assessed included overall and ICU length of stay (LOS), pneumonia severity index (PSI) score on admission, in-hospital mortality, and need for noninvasive positive pressure ventilation as well as supplemental oxygen during admission. Analyses for outcomes were completed by biological sex, sex and age, age within the same sex groups, and sex and primary admission diagnosis. For patients who were alive at discharge, charts were reviewed for the need for supplemental oxygen and ventilation on discharge.

### Data sources

All data were obtained from the Epic Systems (Verona, Wisconsin) EMR retrospectively. Data collection included patient clinical data, laboratory and imaging results, and clinician notes where necessary for determining whether cultures met the inclusion criteria. All chart reviews were completed by trained medical staff. Data were stored using the Research Electronic Data Capture (REDCap) data management platform (31, 32).

### Statistical analyses

Demographic and risk factor characteristics were assessed for differences by biological sex using the Pearson χ test. Continuous variables, including outcome data, were analyzed using a parametric Student t-test or non-parametric Mann–Whitney test, respectively. Multivariate analysis with logistic regression was then performed for select outcomes using covariates including biological sex, age, body mass index (BMI), smoking within 30 days prior to admission, alcohol use within 30 days prior to admission, congestive heart failure status, and chronic obstructive pulmonary disease (COPD) status. For LOS and ICU length of stay (ICU LOS), outcomes were split into two groups depending on whether they were above or below the median value for each outcome. A *P* value less than 0.05 was considered significant. All univariate analyses were performed using GraphPad Prism 10 (GraphPad, La Jolla, CA, USA), and multivariable statistical analysis was performed using R version 4.3.0 (R Foundation for Statistical Computing, Vienna, Austria).

## RESULTS

We identified 1,433 incidences of patients with *A. baumannii* cultures, of which 475 were obtained from respiratory sources (Fig. 1). Of those, 230 cultures were repeat cultures obtained after the initial culture for a patient. In total, 245 cases underwent review, of which 220 met inclusion criteria. By source, 61% were sputum cultures, 34% were bronchoalveolar lavage cultures, and 5% were from tracheal aspirates.

Demographic characteristics are summarized in Table 1, while comorbidities and risk factors are summarized in Table 2. Overall, patients had a median age of 55 (interquartile range [IQR] 27) years without significant differences between males and females (*P* = 0.380). The majority of patients were identified as Black or African American (53%), 30% as Other/Unknown, 15% as White, 1% as Asian, 0.5% as Multiracial, and 0.5% as Native Hawaiian or Other Pacific Islander. By sex, male patients were more likely to be identified as Other/Unknown race (35% vs 22%, *P* = 0.045) and Other/Unknown ethnicity (8% vs 1%, *P* = 0.033), while females were more likely to be identified as not Hispanic or Latino ethnicity (84% vs 69%, *P* = 0.012). Male patients were more likely to have a hospital-acquired *A. baumannii* infection than females (89% vs 74%, *P* = 0.004). Male patients were less likely to be infected with an antimicrobial-resistant strain of *A. baumannii* (55% vs 71%, *P* = 0.021), although there was no significant difference by sex for MDR infections between males and females (45% vs 56%, *P* = 0.109). No other significant demographic deviations were observed (Table 1). Male patients were more likely to have evidence of co-infection on respiratory cultures; 71% of males had other organisms growing on respiratory cultures compared to 48% of females (*P* = 0.0005, Table S1). The coronavirus disease 2019 (COVID-19) pandemic began in 2019, but there was no difference during the study period for male vs female patients in developing COVID-19 (10% vs 7%, *P* = 0.481), requiring intubation (96% vs 93%, *P* = 0.348), or requiring ventilation (95% vs 94%, *P* = 0.746). The most common *A. baumannii*-specific antimicrobial agents used to treat patients meeting the inclusion criteria were meropenem (128 patients, 58.2%), piperacillin–tazobactam (157 patients, 71.4%), ceftriaxone (77 patients, 35.0%), and cefepime (119 patients, 54.1%). In addition, a portion of patients required treatment with last-resort antibiotics (33), including colistin (59 patients, 26.8%), polymyxin B (23 patients, 10.4%), and tigecycline (42 patients, 19.1%; Table S2).

**TABLE 1:** Demographics

Parameter	Total (*n* = 220)	Male (*n* = 138)	Female (*n* = 82)	*P*-value^*a*^
Age (years), median (IQR)	55 (27)	54 (26)	57 (29)	0.380
Race, *n* (%)				
Black or African American	116 (53)	67 (49)	49 (60)	0.107
Other/unknown/not reported	66 (30)	48 (35)	18 (22)	0.045
White	34 (15)	21 (15)	13 (16)	0.900
Asian	2 (1)	0 (0)	2 (2)	0.065
More than one race	1 (0.5)	1 (1)	0 (0)	0.440
Native Hawaiian or other Pacific Islander	1 (0.5)	1 (1)	0 (0)	0.440
Ethnicity, *n* (%)				
Not Hispanic or Latino	164 (75)	95 (69)	69 (84)	0.012
Hispanic or Latino	44 (20)	32 (23)	12 (15)	0.125
Other/unknown/not reported	12 (5)	11 (8)	1 (1)	0.033
BMI, median (IQR)	27 (8)	27 (7)	28 (9)	0.192
Smoking within 30 days of admission, *n* (%)				0.057
Yes	65 (30)	47 (34)	18 (22)	
No	155 (70)	91 (66)	64 (78)	
Alcohol within 30 days of admission, *n* (%)				0.132
Yes	67 (30)	47 (34)	20 (24)	
No	153 (70)	91 (66)	62 (76)	
IVDU within 30 days of admission, *n* (%)				0.183
Yes	15 (7)	7 (5)	8 (10)	
No	205 (93)	131 (95)	74 (90)	
Infection origin status, *n* (%)				0.004
Hospital acquired	184 (84)	123 (89)	61 (74)	
Community acquired	34 (16)	15 (11)	21 (26)	
Culture with resistance to at least one antimicrobial, *n* (%)				0.021
Yes	134 (61)	76 (55)	58 (71)	
No	86 (39)	62 (45)	24 (29)	
Culture with multidrug resistance, *n* (%)				0.109
Yes	108 (49)	62 (45)	46 (56)	
No	112 (51)	76 (55)	36 (44)	
COVID-19 during admission, *n* (%)				0.481
Yes	20 (9)	14 (10)	6 (7)	
No	200 (91)	124 (90)	76 (93)	
Intubation during admission, *n* (%)				0.348
Yes	208 (95)	132 (96)	76 (93)	
No	12 (5)	6 (4)	6 (7)	
Ventilator during admission, *n* (%)				0.746
Yes	208 (95)	131 (95)	77 (94)	
No	12 (5)	7 (5)	5 (6)	

^*a*^ For race and ethnicity, each *P*-value corresponds to the category compared to all other categories combined. Each *P*-value compares males and females for the same category.

**TABLE 2:** Risk factors and comorbidities

Parameter	Total (*n* = 220)	Male (*n* = 138)	Female (*n* = 82)	*P*-value^*a*^
Diabetes, *n* (%)				0.609
Yes	58 (26)	38 (28)	20 (24)	
No	162 (74)	100 (72)	62 (76)	
Cerebrovascular disease, *n* (%)				0.513
Yes	62 (28)	41 (30)	21 (26)	
No	158 (72)	97 (70)	61 (74)	
Dementia, *n* (%)				0.756
Yes	7 (3)	4 (3)	3 (4)	
No	213 (97)	134 (97)	79 (96)	
CHF, *n* (%)				0.513
Yes	28 (13)	16 (12)	12 (15)	
No	192 (87)	122 (88)	70 (85)	
CAD or other HF, *n* (%)				0.740
Yes	30 (14)	18 (13)	12 (15)	
No	190 (86)	120 (87)	70 (85)	
COPD, *n* (%)				0.323
Yes	51 (23)	29 (21)	22 (27)	
No	169 (77)	109 (79)	60 (73)	
Liver disease, *n* (%)				0.926
Yes	22 (10)	14 (10)	8 (10)	
No	198 (90)	124 (90)	74 (90)	
Renal disease, *n* (%)				0.998
Yes	59 (27)	37 (27)	22 (27)	
No	161 (73)	101 (73)	60 (73)	
Hemodialysis, *n* (%)				0.013
Yes	11 (5)	3 (2)	8 (10)	
No	209 (95)	135 (98)	74 (90)	
Malignancy, *n* (%)				0.460
Yes	25 (11)	14 (10)	11 (13)	
No	195 (89)	124 (90)	71 (87)	
HIV/AIDS, *n* (%)				0.064
Yes	11 (5)	4 (3)	7 (8)	
No	209 (95)	134 (97)	75 (92)	
Psychiatric disorder, *n* (%)				0.006
Yes	30 (14)	12 (9)	18 (22)	
No	190 (87)	126 (91)	64 (78)	
Post-surgery, *n* (%)				0.496
Yes	65 (30)	43 (31)	22 (27)	
No	155 (70)	95 (69)	60 (73)	
Hypertension, *n* (%)				0.132
Yes	117 (53)	68 (49)	49 (60)	
No	103 (47)	70 (51)	33 (40)	
Prior antimicrobial use within 90 days of admission, *n* (%)				
Yes	33 (15)	14 (10)	19 (23)	0.009
No	174 (79)	117 (85)	57 (70)	0.007
Unknown	13 (6)	7 (5)	6 (7)	0.495
Influenza within 14 days of admission, *n* (%)				0.595
Yes	4 (2)	2 (1)	2 (2)	
No	216 (98)	136 (99)	90 (98)	
Facility^*b*^ within 90 days of admission, *n* (%)				0.283
Yes	28 (13)	15 (11)	13 (16)	
No	192 (87)	123 (89)	69 (74)	
Prior hospitalization >2 days within 90 days of admission, *n* (%)				0.041
Yes	41 (19)	20 (14)	21 (26)	
No	179 (81)	118 (86)	61 (74)	
MDR organism isolated within last year before admission, *n* (%)				0.269
Yes	7 (3)	3 (2)	4 (5)	
No	213 (97)	135 (98)	78 (95)	

^*a*^ For prior antimicrobial use, each *P*-value corresponds to the category compared to all other categories combined. Each *P*-value compares males and females for the same category.

^*b*^ Facilities were defined as either a nursing home, skilled nursing facility, or long-term acute care facility.

Patient outcomes by sex alone are summarized in Table 3; outcomes from multivariate analysis are summarized in Table S3. Male patients had a longer median LOS compared to females, at 32 days (IQR 27) compared to 24 days (IQR 34) (*P* = 0.0471), respectively. For patients admitted to the ICU, males had a longer median LOS at 23 days (IQR 20) compared to 17 days (IQR 15) (*P* = 0.0009) for females. On multivariate analysis, males were more likely to have prolonged LOS (OR 1.98, 95% CI 1.11–3.55, *P* = 0.0209) and ICU LOS (OR 2.33, 95% CI 1.29–4.29, *P* = 0.00585). There was no difference in mortality at discharge for male vs female patients (30% vs 26%, *P* = 0.444); multivariate analysis did not detect any significant difference between males and females for mortality at discharge (OR 1.48, 95% CI 0.79–2.88, *P* = 0.23). Overall, 94% of the patients were admitted to the ICU (*P* = 0.49) with a median PSI score of 113 (IQR 52; *P* = 0.17). On multivariate analysis, BMI was positively correlated with ICU admission (OR 1.19, 95% CI 1.06–1.36, *P* = 0.00776). Almost all (99%) of the patients required supplemental oxygen during admission, and 13% required noninvasive positive pressure ventilation during admission. There was no significant difference by sex for patients requiring treatment with polymyxin B (females 10% vs males 11%), colistin (females 30% vs males 25%), or tigecycline (females 21% vs males 18%, Table S4). Overall, in both general and ICU settings, males exhibited significant increases in LOS.

**TABLE 3:** Outcomes

Parameter	Total (*n* = 220)	Male (*n* = 138)	Female (*n* = 82)	*P*-value^*a*^
Patient admitted to ICU, *n* (%)				0.495
Yes	207 (94)	131 (95)	76 (93)	
No	13 (6)	7 (5)	6 (7)	
LOS in days, median (IQR)	29 (29)	32 (27)	24 (34)	0.047
ICU LOS in days, median (IQR)	21 (19)	23 (20)	17 (15)	0.001
PSI score, median (IQR)	113 (52)	114 (42)	108 (67)	0.171
Patient required non-invasive positive pressure ventilation (NIPPV) during admission, *n* (%)				
Yes	28 (13)	13 (9.4)	15 (18)	0.056
No	7 (3)	3 (2.2)	4 (5)	0.269
N/A (patient intubated or ventilated)	185 (84)	122 (88.4)	63 (77)	0.023
Patient required supplemental oxygen during admission, *n* (%)				0.273
Yes	218 (99)	136 (99)	82 (100)	
No	2 (1)	2 (1)	0 (0)	
Hospital discharge status, *n* (%)				0.444
Alive	157 (71)	96 (70)	61 (74)	
Deceased	63 (29)	42 (30)	21 (26)	
Patient required supplemental oxygen on discharge, *n* (%)				
Yes	78 (35)	44 (32)	34 (41)	0.151
No	79 (36)	52 (38)	27 (33)	0.477
N/A (patient deceased)	63 (29)	42 (30)	21 (26)	0.444
Type of oxygen received on discharge, *n* (%)				
Invasive (intubation, tracheostomy)	63 (29)	38 (28)	25 (30)	0.640
High flow	4 (2)	2 (1)	2 (2)	0.595
Low flow	11 (5)	4 (3)	7 (9)	0.064
N/A (patient deceased or not on oxygen)	142 (64)	94 (68)	48 (59)	0.151
Patient required ventilation on discharge, *n* (%)				0.666
Yes	33 (15)	19 (14)	14 (17)	0.507
No	124 (56)	77 (56)	47 (57)	0.826
N/A (patient deceased)	63 (29)	42 (30)	21 (26)	0.444

^*a*^ For NIPPV, supplemental oxygen on discharge, type of oxygen on discharge, and ventilation on discharge, each *P*-value corresponds to the category compared to all other categories combined. Each *P*-value compares males and females for the same category.

We next stratified patient outcomes by age (18–50 vs >50 years) for males vs females; these are summarized in Table 4. Males aged 18–50 years had a higher average PSI score compared to females (100 ± 30 vs 79 ± 37, *P* = 0.038); however, there was no difference in mean PSI score for adults >50 years (Table 4). Median LOS overall and for the ICU were similar for males and females aged 18–50 years; however, we did observe that median ICU LOS was longer for males >50 years at 23 days (IQR 22) compared to females at 14 days (IQR 16) (*P* = 0.0099) (Table 4). Females older than 50 years were more likely to require non-invasive positive pressure ventilation (NIPPV) during admission than males (21% vs 9%, *P* = 0.048) and were more likely to receive low-flow oxygen than males older than 50 years on discharge (13% vs 4%, *P* = 0.043). These data indicate that differences between males and females were also evident irrespective of age.

**TABLE 4:** Outcomes stratified by age

Parameter	Males 18–50 years (*n* = 58)	Females 18–50 years (*n* = 29)	*P*-value	Males >50 years (*n* = 80)	Females >50 years (*n* = 53)	*P*-value^*a*^
Patient admitted to ICU, *n* (%)			0.213			0.992
Yes	57 (98)	27 (93)		74 (92)	49 (92)	
No	1 (2)	2 (7)		6 (8)	4 (8)	
LOS in days, median (IQR)	36 (28)	27 (34)	0.880	28 (23)	22 (30)	0.999
ICU LOS in days, median (IQR)	24 (14)	21 (16)	0.999	23 (22)	14 (16)	0.010
PSI score, mean ± SD	100 ± 30	79 ± 37	0.038	129 ± 38	126 ± 44	0.664
Patient required NIPPV during admission, *n* (%)						
Yes	6 (10)	4 (14)	0.635	7 (9)	11 (20.7)	0.048
No	1 (2)	1 (3)	0.613	2 (2)	3 (5.7)	0.348
N/A (patient intubated or on ventilator)	51 (88)	24 (83)	0.510	71 (89)	39 (73.6)	0.024
Patient required supplemental oxygen during admission, *n* (%)			1			0.246
Yes	58 (100)	29 (100)		78 (98)	53 (100)	
No	0 (0)	0 (0)		2 (2)	0 (0)	
Hospital discharge status, *n* (%)			0.719			0.419
Alive	44 (76)	23 (79)		52 (65)	38 (72)	
Deceased	14 (24)	6 (21)		28 (35)	15 (28)	
Patient required supplemental oxygen on discharge, *n* (%)						
Yes	12 (21)	11 (38)	0.086	32 (40)	23 (43.4)	0.697
No	32 (55)	12 (41)	0.225	20 (25)	15 (28.3)	0.672
N/A (patient deceased)	14 (24)	6 (21)	0.719	28 (35)	15 (28.3)	0.419
Type of oxygen received on discharge, *n* (%)						
Invasive (intubation, tracheostomy)	10 (17)	10 (34.5)	0.072	28 (35)	15 (28)	0.419
High flow	1 (2)	1 (3.5)	0.613	1 (1)	1 (2)	0.768
Low flow	1 (2)	0 (0)	0.477	3 (4)	7 (13)	0.043
N/A (patient deceased or not on oxygen)	46 (79)	18 (62)	0.086	48 (60)	30 (57)	0.697
Patient required ventilation on discharge, *n* (%)						
Yes	4 (7)	6 (20.7)	0.057	15 (19)	8 (15)	0.585
No	40 (69)	17 (58.6)	0.339	37 (46)	30 (57)	0.242
N/A (patient deceased)	14 (24)	6 (20.7)	0.719	28 (35)	15 (28)	0.419

^*a*^ For NIPPV, supplemental oxygen on discharge, type of oxygen on discharge, and ventilation on discharge, each *P*-value corresponds to the category compared to all other categories combined. Each *P*-value compares males and females for the same category.

Patient outcomes were then stratified within same-sex groups by age (Table 5). Both males and females in the >50 years age group had higher median PSI scores than their younger counterparts in the 18–50 years age group at 129 ± 38 vs 100 ± 30 (*P* < 0.0001) and 126 ± 44 vs 79 ± 37 (*P* < 0.0001), respectively. Males older than 50 were also more likely to require supplemental oxygen (40% vs 21%, *P* = 0.016) and ventilation (19% vs 7%, *P* = 0.046) on discharge than males 18–50 years. By type of oxygen received on discharge, males older than 50 years were more likely to receive invasive ventilation than males 18–50 (35% vs 17%, *P* = 0.021) years, and females older than 50 years were more likely to receive low-flow oxygen than females 18–50 (13% vs 0%, *P* = 0.041) years.

**TABLE 5:** Outcomes stratified by age within the same-sex group

Parameter	Males 18–50 years (*n* = 58)	Males >50 years (*n* = 80)	*P*-value	Females 18–50 years (*n* = 29)	Females >50 years (*n* = 53)	*P*-value^*a*^
Patient admitted to ICU, *n* (%)			0.127			0.914
Yes	57 (98)	74 (92)		27 (93)	49 (92)	
No	1 (2)	6 (8)		2 (7)	4 (8)	
LOS in days, median (IQR)	36 (28)	28 (23)	0.074	27 (34)	22 (30)	0.999
ICU LOS in days, median (IQR)	24 (14)	23 (22)	0.999	21 (16)	14 (16)	0.430
PSI score, mean ± SD	100 ± 30	129 ± 38	0.0001	79 ± 37	126 ± 44	0.0001
Patient required NIPPV during admission, *n* (%)						
Yes	6 (10)	7 (9)	0.752	4 (14)	11 (20.7)	0.436
No	1 (2)	2 (2)	0.758	1 (3)	3 (5.7)	0.657
N/A (patient intubated or on ventilator)	51 (88)	71 (89)	0.882	24 (83)	39 (73.6)	0.347
Patient required supplemental oxygen during admission, *n* (%)			0.225			1
Yes	58 (100)	78 (98)		29 (100)	53 (100)	
No	0 (0)	2 (2)		0 (0)	0 (0)	
Hospital discharge status, *n* (%)			0.171			0.450
Alive	44 (76)	52 (65)		23 (79)	38 (72)	
Deceased	14 (24)	28 (35)		6 (21)	15 (28)	
Patient required supplemental oxygen on discharge, *n* (%)						
Yes	12 (21)	32 (40)	0.016	11 (38)	23 (43.4)	0.631
No	32 (55)	20 (25)	0.0003	12 (41)	15 (28.3)	0.228
N/A (patient deceased)	14 (24)	28 (35)	0.171	6 (21)	15 (28.3)	0.450
Type of oxygen received on discharge, *n* (%)						
Invasive (intubation, tracheostomy)	10 (17)	28 (35)	0.021	10 (34)	15 (28)	0.561
High flow	1 (2)	1 (1)	0.818	1 (4)	1 (2)	0.661
Low flow	1 (2)	3 (4)	0.484	0 (0)	7 (13)	0.041
N/A (patient deceased or not on oxygen)	46 (79)	48 (60)	0.016	18 (62)	30 (57)	0.631
Patient required ventilation on discharge, *n* (%)						
Yes	4 (7)	15 (19)	0.046	6 (21)	8 (15)	0.520
No	40 (69)	37 (46)	0.008	17 (58)	30 (57)	0.431
N/A (patient deceased)	14 (24)	28 (35)	0.171	6 (21)	15 (28)	0.450

^*a*^ For NIPPV, supplemental oxygen on discharge, type of oxygen on discharge, and ventilation on discharge, each *P*-value corresponds to the category compared to all other categories combined. All *P*-values are between patients aged 18 and 50 and older than 50 years within the same-sex group.

To determine if our observations were a result of the underlying reason for hospitalization, data were then stratified by primary admission diagnosis and sex. Consistent with our observations of males having worse outcomes, the main diagnosis that was statistically significant was among patients admitted with a respiratory diagnosis. Males had a longer median ICU length of stay than females at 24 days (IQR 32 days) compared to 13 days (IQR 41 days; *P* = 0.011), respectively. While median LOS was longer for males at 28 days (IQR 28 days) than females at 15 days (IQR 28 days), the finding was not statistically significant (*P* = 0.259). Male patient outcomes were either worse than or similar to female patient outcomes for all other primary diagnosis groups (Table 6).

**TABLE 6:** Outcomes by sex stratified by primary diagnosis

Primary diagnosis	LOS, days, median (IQR)^*a*^	*P*-value	ICU LOS, days, median (IQR^*a*^	*P*-value	Hospital discharge status, *n* (%)		*P*-value
					Alive	Deceased	
Respiratory (*n* = 22)							
Males (*n* = 8)	28 (28)	0.259	24 (32)	0.011	5 (62)	3 (38)	0.933
Females (*n* = 14)	15 (28)		13 (14)		9 (64)	5 (36)	
Sepsis or other infection (*n* = 49)							
Males (*n* = 29)	22 (18)	0.127	18 (15)	0.228	15 (52)	14 (48)	0.821
Females (*n* = 20)	18 (30)		12 (21)		11 (55)	9 (45)	
Trauma or bleeding (*n* = 66)							
Males (*n* = 46)	36 (26)	0.860	24 (18)	0.358	33 (72)	13 (28)	0.481
Females (*n* = 20)	33 (43)		18 (23)		16 (80)	4 (20)	
Malignancy (*n* = 6)							
Males (*n* = 3)	76 (24)	0.064	77 (23)	0.038	2 (67)	1 (33)	0.273
Females (*n* = 3)	27 (4)		15 (7)		3 (100)	0 (0)	
Gastrointestinal (*n* = 17)							
Males (*n* = 11)	44 (54)	0.905	32 (20)	0.996	8 (73)	3 (27)	0.159
Females (*n* = 6)	39 (65)		32 (26)		6 (100)	0 (0)	
Orthopedics (*n* = 14)							
Males (*n* = 12)	46 (18)	0.121	22 (17)	0.670	12 (100)	0 (0)	1.000
Females (*n* = 2)	32 (8)		25 (8)		2 (100)	0 (0)	
Poisoning or other drug toxicity (*n* = 7)							
Males (*n* = 4)	16 (10)	0.305	13 (10)	0.482	2 (50)	2 (50)	0.659
Females (*n* = 3)	28 (16)		19 (11)		2 (67)	1 (33)	
Cardiac (*n* = 16)							
Males (*n* = 12)	29 (18)	0.272	24 (15)	0.270	8 (67)	4 (33)	0.755
Females (*n* = 4)	40 (43)		16 (12)		3 (75)	1 (25)	
Neurologic (*n* = 23)							
Males (*n* = 13)	31 (14)	0.383	28 (10)	0.134	11 (85)	2 (15)	0.704
Females (*n* = 10)	39 (26)		18 (17)		9 (90)	1 (10)	

^*a*^ For primary diagnoses related to malignancy or poisoning/other drug toxicity, the mean LOS and ICU LOS are reported because data are normally distributed. For gastrointestinal primary diagnoses, the mean ICU LOS is reported because the data are normally distributed.

As a final analysis, patient outcomes were stratified by comorbidities and risk factors (Table S5). Patients who had recently undergone surgery (*P* = 0.0014) or who had used alcohol within 30 days prior to admission (*P* = 0.035) had longer overall LOS. Alcohol use within 30 days (*P* = 0.024) and smoking within 30 days of admission (*P* < 0.001) were both associated with longer ICU LOS. Patients with COPD (*P* = 0.024) or renal disease (*P* < 0.001) had higher mortality rates. These findings indicate that a specific set of health conditions and activities adversely affect the outcomes of *A. baumannii* pneumonia.

## DISCUSSION

To our knowledge, this is the first published study examining the relationship between biological sex and clinical outcomes in patients with *A. baumannii* pneumonia. In our study, male patients had worse outcomes than female patients, including a significantly longer overall and ICU LOS. However, overall LOS was similar when stratifying into two age groups, suggesting that this difference may have been driven by the subset of critically ill patients >50 years who required ICU admission. Furthermore, male patients were more likely to have evidence of co-infection on respiratory or blood cultures. For patients 18–50 years, males had higher PSI severity scores on admission compared to females. Certain clinical outcomes were worse with age, as older males required more interventions than younger males. There was a high mortality rate as 29% of patients died during their inpatient admission, but there was no significant difference in inpatient mortality between male and female patients. Our study population is also broadly reflective of the Newark population, with comparable racial and ethnic composition to data from the US Census Bureau (34).

Our finding of a male incidence of 63% in our cohort is comparable to other retrospective studies conducted in China and the European Union, where incidence reaches up to 74% for male patients (27–29). Another study incidentally noted that 93% of patients living with HIV who had a comorbid *A. baumannii* infection were male (35). In this study’s cohort, the average overall LOS for patients with HIV and *A. baumannii* was 40.3 days (SD 28.3), much longer than the median LOS for patients with HIV/AIDS and *A. baumannii* in our study at 15 days (IQR 20 days) (35). Our findings contrast with prior work using mice, which demonstrated that female C57BL/6J mice had higher mortality rates and bacterial burden compared to male mice (26). In the setting of a higher prevalence of co-infection in our study, the mechanism underpinning this difference and the specific pathogenesis in *A. baumannii* infection requires further, rigorous investigation. Possible mechanisms explaining these differences might include hormonal and environmental factors, microbiome-mediated interactions that have different effects in mice compared to humans, or behavior patterns that differ according to sex. As discussed, with other bacterial pathogens, such as *Staphylococcus aureus*, *A. baumannii* has adapted to selection pressure from the human immune system, so humanized mice models may have increased utility in understanding the mechanisms underlying the difference in sex bias with regard to mortality and morbidity of infection in humans vs mice (36). In our study, we found that male patients were more likely to have hospital-acquired *A. baumannii* infections than females; hospital-acquired infections typically exhibit worse clinical outcomes than community-acquired infections and are associated with high levels of antimicrobial resistance (37, 38). However, in our study population, females had a higher rate of antimicrobial resistance and multidrug resistance, and furthermore, there was no difference in the requirements for last-resort antibiotics by sex. When adjusting for the underlying reason for hospitalization, only the respiratory primary diagnosis group showed a longer ICU length of stay for males compared to females that met statistical significance. Mortality rates were increased for patients with COPD and renal disease, while alcohol use or smoking within 30 days prior to admission or recent surgery all negatively impacted LOS. COPD is the sixth leading cause of death in the United States, and our findings align with the literature showing that men are more likely to die from COPD than women (39). Our findings reflect the general consensus that mortality among female patients with chronic kidney disease is lower than in males, including within the US across all ages, but the opposite is true in several countries including Afghanistan and Algeria (40). Recent surgery, alcohol use, and smoking are all generally associated with worse patient outcomes, including prolonged LOS (41, 42), though other studies have failed to associate recent alcohol use or smoking with prolonged LOS in patients with pneumonia (43, 44).

One limitation of this study is that some patients had multiple ICU admissions because of transfers in and out of the unit, so their overall ICU LOS may be underestimated. As a retrospective, single-center study, the generalizability of our results is limited. Future studies on sex differences should ideally include patients from multiple centers and should have a prospective design to permit more thorough analysis of laboratory data including cell counts for neutrophils and alveolar macrophages. These data would be crucial for further investigation of the mechanism driving sex differences in infection severity and outcomes.

### Conclusions

Despite the recognition of *A. baumannii* infections as a serious global healthcare threat, understanding of how biological sex impacts their course in humans is limited. In contrast to previous work in mice, we show that male sex is associated with longer overall and ICU LOS in patients with *A. baumannii* pneumonia. We also demonstrate that COPD and renal disease are associated with increased mortality rates in *A. baumannii* patients, while recent surgery, alcohol use, or smoking increased LOS. This study determines that men, the elderly, and patients with certain underlying comorbidities and risk factors have worse outcomes in *A. baumannii* pneumonia, which can guide the clinical management of these patients during inpatient admission.

## SUPPLEMENTAL MATERIAL

Table S1 spectrum.03199-24-s0001.docx Respiratory and blood culture co-infection by sex.

Table S2 spectrum.03199-24-s0002.xlsx *Acinetobacter baumannii*-specific antibiotic therapy profile by patient.

Table S3 spectrum.03199-24-s0003.docx Multivariate analysis of patient outcomes.

Table S4 spectrum.03199-24-s0004.docx Last resort antibiotic therapy by sex.

Table S5 spectrum.03199-24-s0005.xlsx Outcomes stratified by comorbidities and risk factors.

ASM does not own the copyrights to Supplemental Material that may be linked to, or accessed through, an article. The authors have granted ASM a non-exclusive, world-wide license to publish the Supplemental Material files. Please contact the corresponding author directly for reuse.

## References

[1] Dunn SE, Perry WA, Klein SL. 2024. Mechanisms and consequences of sex differences in immune responses. Nat Rev Nephrol 20:37–55. doi:10.1038/s41581-023-00787-w 37993681

[2] Klein SL, Flanagan KL. 2016. Sex differences in immune responses. Nat Rev Immunol 16:626–638. doi:10.1038/nri.2016.90 27546235

[3] Scully EP. 2022. Sex, gender and infectious disease. Nat Microbiol 7:359–360. doi:10.1038/s41564-022-01064-5 35246651

[4] Clayton JA. 2016. Studying both sexes: a guiding principle for biomedicine. FASEB J 30:519–524. doi:10.1096/fj.15-279554 26514164 PMC4714546

[5] Mauvais-Jarvis F, Bairey Merz N, Barnes PJ, Brinton RD, Carrero J-J, DeMeo DL, De Vries GJ, Epperson CN, Govindan R, Klein SL, Lonardo A, Maki PM, McCullough LD, Regitz-Zagrosek V, Regensteiner JG, Rubin JB, Sandberg K, Suzuki A. 2020. Sex and gender: modifiers of health, disease, and medicine. Lancet 396:565–582. doi:10.1016/S0140-6736(20)31561-0 32828189 PMC7440877

[6] Klein SL, Marks MA, Li W, Glass GE, Fang LQ, Ma JQ, Cao WC. 2011. Sex differences in the incidence and case fatality rates from hemorrhagic fever with renal syndrome in China, 2004-2008. Clin Infect Dis 52:1414–1421. doi:10.1093/cid/cir232 21628481 PMC3146012

[7] Jacobsen H, Klein SL. 2021. Sex differences in immunity to viral infections. Front Immunol 12:720952. doi:10.3389/fimmu.2021.720952 34531867 PMC8438138

[8] Murphy G, Pfeiffer R, Camargo MC, Rabkin CS. 2009. Meta-analysis shows that prevalence of Epstein-Barr virus-positive gastric cancer differs based on sex and anatomic location. Gastroenterology 137:824–833. doi:10.1053/j.gastro.2009.05.001 19445939 PMC3513767

[9] Wang SH, Yeh SH, Lin WH, Wang HY, Chen DS, Chen PJ. 2009. Identification of androgen response elements in the enhancer I of hepatitis B virus: a mechanism for sex disparity in chronic hepatitis B. Hepatology 50:1392–1402. doi:10.1002/hep.23163 19670412

[10] Fleming DT, McQuillan GM, Johnson RE, Nahmias AJ, Aral SO, Lee FK, St Louis ME. 1997. Herpes simplex virus type 2 in the United States, 1976 to 1994. N Engl J Med 337:1105–1111. doi:10.1056/NEJM199710163371601 9329932

[11] Griesbeck M, Altfeld M. 2015. Sex differences in the manifestations of HIV-1 infection, p 103–181. In Sex and gender differences in infection and treatments for infectious diseases

[12] Garenne M. 1994. Sex differences in measles mortality: a world review. Int J Epidemiol 23:632–642. doi:10.1093/ije/23.3.632 7960393

[13] Bowen VB, Braxton J, Davis DW, Flagg EW, Grey J, Grier L, Harvey A, Kidd S, Kreisel K, Llata E. 2019. Sexually transmitted disease surveillance 2018

[14] Anonymous. 2019. Gonorrhea. Annual epidemiological report for 2018. In European Centre for Disease Prevention and Control. ECDC, Stockholm, Sweden.

[15] Ong JJ, Fethers K, Howden BP, Fairley CK, Chow EPF, Williamson DA, Petalotis I, Aung E, Kanhutu K, De Petra V, Chen MY. 2017. Asymptomatic and symptomatic urethral gonorrhoea in men who have sex with men attending a sexual health service. Clin Microbiol Infect 23:555–559. doi:10.1016/j.cmi.2017.02.020 28257898

[16] Dias SP, Brouwer MC, van de Beek D. 2022. Sex and gender differences in bacterial infections. Infect Immun 90:e0028322. doi:10.1128/iai.00283-22 36121220 PMC9584217

[17] Anonymous. 2020. Chlamydia infection. Annual epidemiological report for 2018. In European Centre for Disease Prevention and Control. ECDC, Stockholm, Sweden.

[18] Ngun TC, Ghahramani N, Sánchez FJ, Bocklandt S, Vilain E. 2011. The genetics of sex differences in brain and behavior. Front Neuroendocrinol 32:227–246. doi:10.1016/j.yfrne.2010.10.001 20951723 PMC3030621

[19] Akoolo L, Pires S, Kim J, Parker D. 2022. The capsule of Acinetobacter baumannii protects against the innate immune response. J Innate Immun 14:543–554. doi:10.1159/000522232 35320810 PMC9485954

[20] Antunes LCS, Visca P, Towner KJ. 2014. Acinetobacter baumannii: evolution of a global pathogen. Pathog Dis 71:292–301. doi:10.1111/2049-632X.12125 24376225

[21] Zampaloni C, Mattei P, Bleicher K, Winther L, Thäte C, Bucher C, Adam J-M, Alanine A, Amrein KE, Baidin V, et al.. 2024. A novel antibiotic class targeting the lipopolysaccharide transporter. Nature 625:566–571. doi:10.1038/s41586-023-06873-0 38172634 PMC10794144

[22] Dijkshoorn L, Nemec A, Seifert H. 2007. An increasing threat in hospitals: multidrug-resistant Acinetobacter baumannii. Nat Rev Microbiol 5:939–951. doi:10.1038/nrmicro1789 18007677

[23] Tacconelli E, Carrara E, Savoldi A, Harbarth S, Mendelson M, Monnet DL, Pulcini C, Kahlmeter G, Kluytmans J, Carmeli Y, Ouellette M, Outterson K, Patel J, Cavaleri M, Cox EM, Houchens CR, Grayson ML, Hansen P, Singh N, Theuretzbacher U, Magrini N, WHO Pathogens Priority List Working Group. 2018. Discovery, research, and development of new antibiotics: the WHO priority list of antibiotic-resistant bacteria and tuberculosis. Lancet Infect Dis 18:318–327. doi:10.1016/S1473-3099(17)30753-3 29276051

[24] Ikuta KS, Swetschinski LR, Robles Aguilar G, Sharara F, Mestrovic T, Gray AP, Davis Weaver N, Wool EE, Han C, Gershberg Hayoon A, et al.. 2022. Global mortality associated with 33 bacterial pathogens in 2019: a systematic analysis for the Global Burden of Disease Study 2019. The Lancet 400:2221–2248. doi:10.1016/S0140-6736(22)02185-7 PMC9763654 36423648

[25] Murray CJL, Ikuta KS, Sharara F, Swetschinski L, Robles Aguilar G, Gray A, Han C, Bisignano C, Rao P, Wool E, et al.. 2022. Global burden of bacterial antimicrobial resistance in 2019: a systematic analysis. The Lancet 399:629–655. doi:10.1016/S0140-6736(21)02724-0 PMC8841637 35065702

[26] Pires S, Peignier A, Seto J, Smyth DS, Parker D. 2020. Biological sex influences susceptibility to Acinetobacter baumannii pneumonia in mice. JCI Insight 5:e132223. doi:10.1172/jci.insight.132223 32191638 PMC7205275

[27] Yuan WL, Shen YJ, Deng DY. 2018. Sex bias of Acinetobacter baumannii nosocomial infection. Am J Infect Control 46:957–958. doi:10.1016/j.ajic.2018.04.231 29910034

[28] Brandl M, Hoffmann A, Willrich N, Reuss A, Reichert F, Walter J, Eckmanns T, Haller S. 2021. Bugs that can resist antibiotics but not men: gender-specific differences in notified infections and colonisations in Germany, 2010-2019. Microorganisms 9:894. doi:10.3390/microorganisms9050894 33922011 PMC8143559

[29] Ayobami O, Willrich N, Suwono B, Eckmanns T, Markwart R. 2020. The epidemiology of carbapenem-non-susceptible Acinetobacter species in Europe: analysis of EARS-Net data from 2013 to 2017. Antimicrob Resist Infect Control 9:89. doi:10.1186/s13756-020-00750-5 32560670 PMC7304165

[30] Magiorakos AP, Srinivasan A, Carey RB, Carmeli Y, Falagas ME, Giske CG, Harbarth S, Hindler JF, Kahlmeter G, Olsson-Liljequist B, Paterson DL, Rice LB, Stelling J, Struelens MJ, Vatopoulos A, Weber JT, Monnet DL. 2012. Multidrug-resistant, extensively drug-resistant and pandrug-resistant bacteria: an international expert proposal for interim standard definitions for acquired resistance. Clin Microbiol Infect 18:268–281. doi:10.1111/j.1469-0691.2011.03570.x 21793988

[31] Harris P.A, Taylor R, Thielke R, Payne J, Gonzalez N, Conde JG. 2009. Research electronic data capture (REDCap)--a metadata-driven methodology and workflow process for providing translational research informatics support. J Biomed Inform 42:377–381. doi:10.1016/j.jbi.2008.08.010 18929686 PMC2700030

[32] Harris PA, Taylor R, Minor BL, Elliott V, Fernandez M, O’Neal L, McLeod L, Delacqua G, Delacqua F, Kirby J, Duda SN, REDCap Consortium. 2019. The REDCap consortium: building an international community of software platform partners. J Biomed Inform 95:103208. doi:10.1016/j.jbi.2019.103208 31078660 PMC7254481

[33] Vázquez-López R, Solano-Gálvez SG, Juárez Vignon-Whaley JJ, Abello Vaamonde JA, Padró Alonzo LA, Rivera Reséndiz A, Muleiro Álvarez M, Vega López EN, Franyuti-Kelly G, Álvarez-Hernández DA, Moncaleano Guzmán V, Juárez Bañuelos JE, Marcos Felix J, González Barrios JA, Barrientos Fortes T. 2020. Acinetobacter baumannii resistance: a real challenge for clinicians. Antibiotics (Basel) 9:205. doi:10.3390/antibiotics9040205 32340386 PMC7235888

[34] Anonymous. 2024. QuickFacts: Newark City, New Jersey. United States Census. Available from: https://www.census.gov/quickfacts/fact/table/newarkcitynewjersey

[35] Yang J, Tang Q, Qi T, Chen J, Ji Y, Tang Y, Wang Z, Song W, Xun J, Liu L, Shen Y, Zhang R, Lu H. 2018. Characteristics and outcomes of Acinetobacter baumannii infections in patients with HIV: a matched case-control study. Sci Rep 8:15617. doi:10.1038/s41598-018-33753-9 30353067 PMC6199303

[36] Parker D. 2017. Humanized mouse models of Staphylococcus aureus infection. Front Immunol 8:512. doi:10.3389/fimmu.2017.00512 28523002 PMC5415562

[37] Rothberg MB, Haessler S, Lagu T, Lindenauer PK, Pekow PS, Priya A, Skiest D, Zilberberg MD. 2014. Outcomes of patients with healthcare-associated pneumonia: worse disease or sicker patients? Infect Control Hosp Epidemiol 35 Suppl 3:S107–15. doi:10.1086/677829 25222889 PMC4559081

[38] Mehrad B, Clark NM, Zhanel GG, Lynch JP III. 2015. Antimicrobial resistance in hospital-acquired gram-negative bacterial infections. Chest 147:1413–1421. doi:10.1378/chest.14-2171 25940252 PMC4420185

[39] Anonymous. 2021. COPD trends brief: mortality. American Lung Association.

[40] Hockham C, Schanschieff F, Woodward M. 2022. Sex differences in CKD-associated mortality from 1990 to 2019: data from the Global Burden of Disease Study. Kidney Med 4:100535. doi:10.1016/j.xkme.2022.100535 36159166 PMC9490202

[41] Marfil-Garza BA, Belaunzarán-Zamudio PF, Gulias-Herrero A, Zuñiga AC, Caro-Vega Y, Kershenobich-Stalnikowitz D, Sifuentes-Osornio J. 2018. Risk factors associated with prolonged hospital length-of-stay: 18-year retrospective study of hospitalizations in a tertiary healthcare center in Mexico. PLoS One 13:e0207203. doi:10.1371/journal.pone.0207203 30408118 PMC6224124

[42] Millett ERC, De Stavola BL, Quint JK, Smeeth L, Thomas SL. 2015. Risk factors for hospital admission in the 28 days following a community-acquired pneumonia diagnosis in older adults, and their contribution to increasing hospitalisation rates over time: a cohort study. BMJ Open 5:e008737. doi:10.1136/bmjopen-2015-008737 PMC4679905 26631055

[43] Menéndez R, Cremades MJ, Martínez-Moragón E, Soler JJ, Reyes S, Perpiñá M. 2003. Duration of length of stay in pneumonia: influence of clinical factors and hospital type. Eur Respir J 22:643–648. doi:10.1183/09031936.03.00026103 14582918

[44] Alfares M, Almrzouqi A, Alghamdi R, Alsharif R, Kurdi L, Kamfar S, Alzahrani F, Maimani L. 2023. Risk factors of hospital-acquired pneumonia among hospitalized patients with cardiac diseases. Cureus 15:e34253. doi:10.7759/cureus.34253 36726767 PMC9886362

