# Supplementary material for: Biological sex influences severity and outcomes in Acinetobacter baumannii pneumonia
Source: Microbiol Spectr. 2025 Apr 16;13(6):e03199-24. doi: 10.1128/spectrum.03199-24 (PMC12131798; doi:10.1128/spectrum.03199-24)
Supplement: Table S1 — Respiratory and blood culture co-infection by sex. [file spectrum.03199-24-s0001.docx]

**Supplemental Table 1. Respiratory and Blood Culture Co-Infection by Sex.**

| **Parameter** | **Total (n=220)** | **Male (n=138)** | **Female (n=82)** | **p-value^*^** |
| --- | --- | --- | --- | --- |
| **Additional organisms on respiratory culture, n (%)** |  | | | 0.0005 |
| Yes | 137 (62) | 98 (71) | 39 (48) |  |
| No | 83 (38) | 40 (29) | 43 (52) |  |
| **Additional organisms on blood culture, n (%)** |  | | | 0.575 |
| Yes | 39 (18) | 26 (19) | 13 (16) |  |
| No | 181 (82) | 112 (82) | 69 (84) |  |
