# Supplementary material for: Biological sex influences severity and outcomes in Acinetobacter baumannii pneumonia
Source: Microbiol Spectr. 2025 Apr 16;13(6):e03199-24. doi: 10.1128/spectrum.03199-24 (PMC12131798; doi:10.1128/spectrum.03199-24)
Supplement: Table S3 — Multivariate analysis of patient outcomes. [file spectrum.03199-24-s0003.docx]

**Supplemental Table 3. Multivariate Analysis of Patient Outcomes.**

| **Parameter** | **Odds Ratio, ICU Admission (95% CI)** | **p-value^*^** |
| --- | --- | --- |
| BMI | 1.19 (1.06-1.36) | 0.00776 |
| **Parameter** | **Odds Ratio, LOS (95% CI)** | **p-value^*^** |
| Male Sex | 1.98 (1.11-3.55) | 0.0209 |
| **Parameter** | **Odds Ratio, ICU LOS (95% CI)** | **p-value^*^** |
| Male Sex | 2.33 (1.29-4.29) | 0.00585 |
| **Parameter** | **Odds Ratio, Mortality Discharge Status (95% CI)** | **p-value^*^** |
| Male Sex | 1.48 (0.79-2.88) | 0.230 |
