# Supplementary material for: Biological sex influences severity and outcomes in Acinetobacter baumannii pneumonia
Source: Microbiol Spectr. 2025 Apr 16;13(6):e03199-24. doi: 10.1128/spectrum.03199-24 (PMC12131798; doi:10.1128/spectrum.03199-24)
Supplement: Table S4 — Last resort antibiotic therapy by sex. [file spectrum.03199-24-s0004.docx]

**Supplemental Table 4. Last Resort Antibiotic Therapy by Sex.**

| **Parameter** | **Total (n=220)** | **Male (n=138)** | | **Female (n=82)** | | **p-value^*^** |
| --- | --- | --- | --- | --- | --- | --- |
| **Patient required treatment with polymyxin, n (%)** |  | | 0.794 | |  |  |
| Yes | 23 (10) | 15 (11) | | 8 (10) | |  |
| No | 197 (90) | 123 (89) | | 74 (90) | |  |
| **Patient required treatment with colistin, n (%)** |  | | 0.343 | |  |  |
| Yes | 59 (27) | 34 (25) | | 25 (30) | |  |
| No | 161 (73) | 104 (75) | | 57 (70) | |  |
| **Patient required treatment with tigecycline, n (%)** |  | | 0.633 | |  |  |
| Yes | 42 (19) | 25 (18) | | 17 (21) | |  |
|  | 178 (81) | 113 (82) | | 65 (79) | |  |
